# Diffuse Peritonitis Caused by Splenic Abscess After Laparoscopic Sleeve Gastrectomy: A Case Report

**DOI:** 10.7759/cureus.46150

**Published:** 2023-09-28

**Authors:** Diogo Silva, Alexandre Alves, Rui F Almeida, Mário Nora

**Affiliations:** 1 General Surgery, Centro Hospitalar de Entre o Douro e Vouga, Santa Maria da Feira, PRT

**Keywords:** emergency surgery, splenectomy, bariatric complication, splenic abscess, sleeve gastrectomy

## Abstract

A splenic abscess (SA) following sleeve gastrectomy (SG) is a rare manifestation of a gastric leak (GL). The clinical findings include fever, abdominal pain, leukocytosis, and an elevated C-reactive protein. A computed tomography scan is diagnostic and can show signs of GL, or diffuse peritonitis. Treatment can either be non-operative (including large-spectrum antibiotics and percutaneous drainage) or surgical (including splenectomy). We present the case of a 41-year-old female patient with SA, with septic shock and diffuse peritonitis, successfully treated with a splenectomy three months post-SG.

## Introduction

Obesity is a global pandemic, with cases involving weight issues having tripled over the past 50 years. Bariatric surgery has shown the best long-term results in treating this disease and the complications that arise from it. Nowadays, sleeve gastrectomy (SG) is the bariatric procedure performed most commonly worldwide (around 58% of all bariatric surgeries) [1]. Initially offered as a first-stage procedure in the surgical management of super-obese patients, it has been commonly used due to its safety and effectiveness in weight loss and comorbidity resolution [2]. However, a gastric leak (GL) is one of its most serious and feared complications, and it can occur in up to 2.4% of cases [3]. Moreover, in the literature, there are several case reports related to splenic abscesses (SAs) caused by GL after SG [4,5].

We present the case of an SA in a patient with GL after SG.

## Case presentation

A 41-year-old woman with a body mass index (BMI) of 42 kg/m^2^ and hypertension underwent laparoscopic SG with an uneventful postoperative recovery.

Three months after surgery, she was admitted to the emergency department with a fever and left upper quadrant (LUQ) abdominal pain without nausea or vomiting. Physical examinations revealed a fever (38.5°C), tachycardia (110 bpm), and systolic blood pressure (less than 90 mmHg). The surgical wounds were healed, and the abdomen was tender in the LUQ. Blood tests revealed white blood cell elevation (14.4) with a left shift, high C-reactive protein (382.8 mg/L), a high procalcitonin level (7.30 ng/ml), and lactic acidosis (2.7 mmol/L) (Table 1).

**Table 1:** The patient's laboratory test results

Variable	Reference value	Upon admission
Hemoglobin (g/dL)	12.0–16.0	10.1
White cell count (10^9^/L)	4.0–11.0	14.4
Platelet count (10^9^/L)	150–450	169
International normalized ratio	1	1.5
Sodium (mmol/L)	136–145	138
Potassium (mmol/L)	3.5–5.1	2.9
Urea (mg/dL)	15–40	31
Creatinine (mg/dL)	0.6–1.1	0.6
Total bilirubin (mg/dL)	0.2–1.2	3.1
Direct bilirubin (mg/dL)	0.0–0.5	2.1
C-reactive protein (mg/L)	< 5.0	382.8
Procalcitonin (ng/mL)	0.0–0.5	7.3
Lactic acid (mmol/L)	< 2.0	2.7

**Figure 1:**
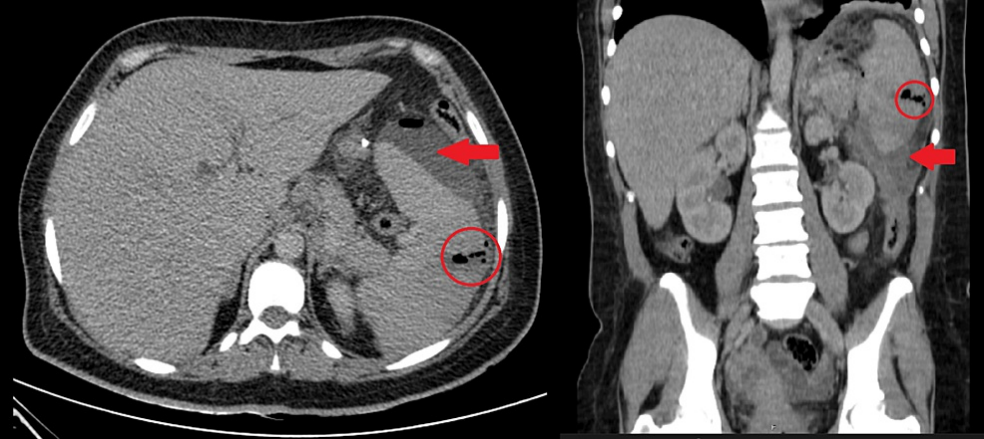
A CT scan upon admission showed a splenic abscess. The spleen abscess is marked with a circle, and the perisplenic free fluid is marked with arrows.

A computed tomography (CT) scan of the abdomen was ordered, and it revealed an LUQ collection of gas and fluid (11.0 × 7.0 × 5.0 cm), free fluid in the peritoneal cavity, and a lobulated area of altered perfusion in the spleen suggesting an abscess (Figure 1).

**Figure 2:**
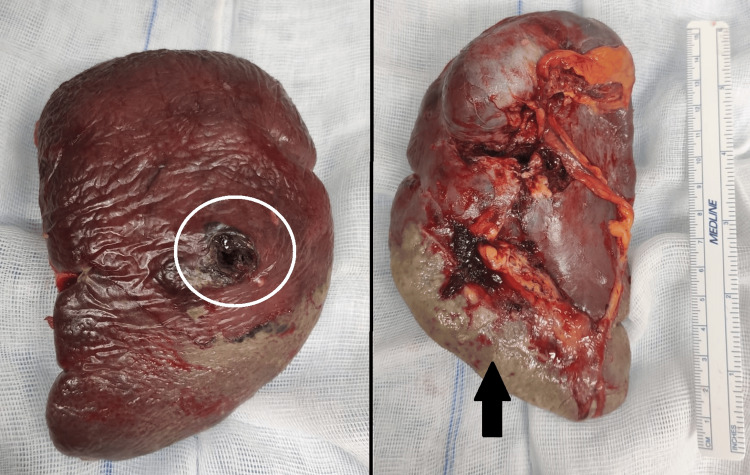
A splenectomy surgical specimen showing an abscess and infarct areas The spleen abscess cavity is marked with a circle, and infarct areas are marked with an arrow.

Intravenous fluid resuscitation and parenteral broad-spectrum antibiotics were started. Emergency surgery was decided. At laparotomy, there was an abscess cavity within the spleen that had ruptured onto the diaphragmatic surface, which caused significant intraperitoneal spillage. A splenectomy and tests of the gastric staple line’s integrity with methylene blue instillation were performed (with no evidence of a leak). Moreover, a drain was left in the subphrenic space. The removed spleen showed an abscess and infarct areas (Figure 2).

**Figure 3:**
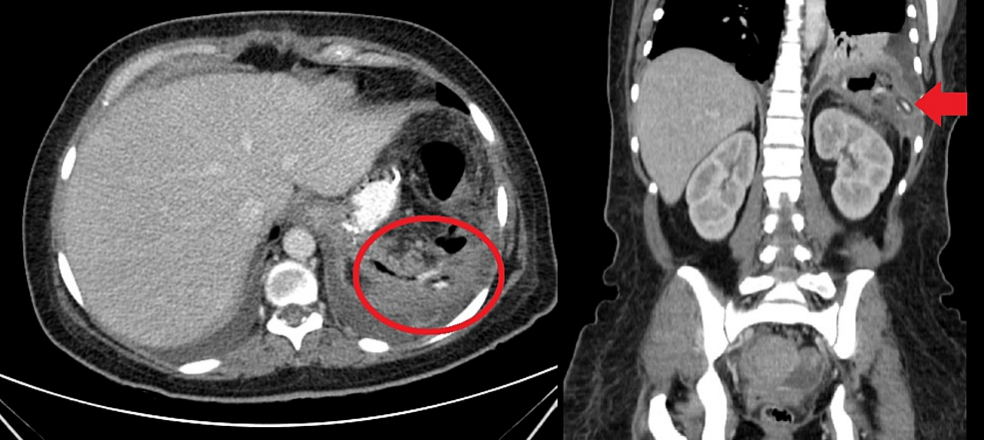
A postoperative (fourth day) CT scan showing contrast inside the drain, a sign of gastric leak The postoperative abscess is marked with a circle, and the contrast inside the drain is marked with an arrow.

Despite showing improvement, the patient displayed suspicious intra-abdominal drainage on the fourth postoperative day. A test involving an oral intake of methylene blue was positive. In addition, an oral and intravenous (IV) contrasted CT scan revealed a left pleural effusion with basal atelectasis and a small collection of contrast near the gastric staple line and inside the drain (confirming a GL) (Figure 3).

Parenteral feeding began with no oral intake. During the following week, clinical improvement was observed, and there was a daily total drainage output of less than 30 ml. On the twelfth postoperative day, esophagogastroduodenoscopy was performed, which showed the presence of luminal constriction (compatible with post-sleeve status) without significant stenosis or leak. Furthermore, oral feeding with clear liquids began. Two weeks after the splenectomy, the patient was symptom-free with vestigial drainage and was discharged after pneumococcal, meningococcal, and *Haemophilus influenzae* vaccinations. On the nineteenth postoperative day, the abdominal drain was removed.

During the six-month period post-SG consultation, the patient was fine and had no complaints. She had lost 37 kg, had a BMI of 27.1 kg/m2 (total weight loss (TWL): 36% and excess weight loss (EWL): 86%), and her blood pressure was under control without medication.

## Discussion

A splenic abscess is a rare entity with a potentially life-threatening evolution characterized by the presence of a fever, diffuse LUQ pain, and left-side pleural effusion [6]. When a patient with a localized case is stable, treatment should consist of large-spectrum antibiotics and percutaneous drainage. However, when signs of instability with septic shock are present, surgery is mandatory [7].

The main causes of SA are bacteriemia, trauma with superinfection of hematoma, immunodeficiency, neoplasms, metastatic infection, parasitic infection, contiguous focus of infection, and splenic infarction [7]. It has also been reported after some invasive procedures like embolization via interventional radiology techniques to treat splenic artery pseudoaneurysm formation [8] or gastric procedures like Nissen fundoplication and gastrectomy for cancer [9].

A splenic abscess after SG is a rare condition, and there are fewer than 15 case reports in the literature [4]. There are multiple mechanisms described for SA formation following SG. They are iatrogenic splenic injury during surgery, splenic ischemia, temporary immune suppression in the immediate postoperative course, and GL with an abscess formation extending to the spleen.

In our case, the patient had severe sepsis with signs of diffuse peritonitis, so surgery was proposed. During surgery, the spleen showed a large area of infarction, and a splenectomy was performed. As we are aware of the possibility of GL following SG, the stapled line was tested with blue methylene, but no extravasation was witnessed. Moreover, we decided to leave a drain, which was of great importance since, in the postoperative period, the patient displayed signs of a leak that were confirmed with an oral contrasted CT scan.

Treatment for GL should include drainage, antibiotics, stopping oral feeding with total parenteral nutrition, and an endoscopic approach using coated self-expandable stents or pigtail catheters. This approach induces positive results related to morbidity and survival [10]. In our case, when the endoscopic evaluation had been conducted (two weeks after surgery), the patient displayed gastric constriction (post-sleeve status) but without significant stenosis (a major cause of a GL following SG), and the leak was no longer present. Therefore, oral feeding began without any drainage observed. As the patient tolerated oral feeding without any complaints, they were discharged, and the drain was removed during the outpatient consultation.

After a splenectomy, attention must be given to postoperative complications, such as infection risks. These include life-threatening sepsis, especially with encapsulated organisms such as *Streptococcus pneumoniae*, *Haemophilus influenzae*, and *Neisseria meningitidis*. Vaccinations against these organisms can reduce risk, which is why our patient was vaccinated on the fourteenth postoperative day.

## Conclusions

A splenic abscess (SA) is a rare manifestation of GL following SG, and our case proves that special attention should be given to bariatric patients. The decision to undergo a splenectomy will be based on clinical status and response to treatment. However, it is mandatory when there are signs of septic shock.
